# Urinary Organic Acid Profiling by GC-MS Reveals Distinct Metabolic Signatures for Non-Invasive Diagnosis and Disease Activity Monitoring of Inflammatory Bowel Disease

**DOI:** 10.3390/ijms27146318

**Published:** 2026-07-16

**Authors:** Emilia Cuencas Barrientos, Leonela Tantucci, Dafne Cabrera, Marina L. Fernandez, Adriana Fochesato, Andrea Delgado, Silvia G. Correa, Domingo C. Balderramo, Pablo A. Romagnoli

**Affiliations:** 1 Centro de Investigación en Medicina Traslacional “Severo R. Amuchástegui” (CIMETSA), Instituto Universitario de Ciencias Biomédicas de Córdoba (IUCBC), U.A. Consejo Nacional de Investigaciones Científicas y Técnicas (CONICET), Córdoba X5016KEJ, Argentina; emilia.cuencas@iucbc.edu.ar; 2 Servicio de Inmunología, Fundación Para el Progreso de la Medicina, Córdoba X5000EMS, Argentina; 3 Servicio de Gastroenterología, Hospital Privado Universitario de Córdoba, Córdoba X5016KEH, Argentina; 4 Laboratorio Central, Hospital Privado Universitario de Córdoba, Córdoba X5016KEH, Argentina; marina.fernandez@iucbc.edu.ar; 5 Metabolómica Lab, Córdoba X5000IUN, Argentina; 6 Centro de Investigaciones en Bioquímica Clínica e Inmunología (CIBICI), Facultad de Ciencias Químicas, Universidad Nacional de Córdoba, Córdoba X5000HVA, Argentina; silviagracielacorrea@unc.edu.ar

**Keywords:** inflammatory bowel disease, urinary metabolomics, non-invasive biomarkers, organic acids, gas chromatography–mass spectrometry, ulcerative colitis, Crohn’s disease

## Abstract

Inflammatory bowel disease (IBD) prevalence is rising rapidly in South America, yet non-invasive diagnostic and monitoring strategies remain inadequate. Urine samples from 30 individuals (10 healthy controls [C], 10 ulcerative colitis [UC] and 10 Crohn’s disease [CD] patients) from a South American cohort were analyzed by gas chromatography–mass spectrometry (GC-MS); metabolomic profiling employed multivariate statistics, pathway enrichment analysis, and receiver operating characteristic (ROC) curve analysis. Profiling of 62 organic acids revealed distinct metabolic patterns across groups: UC patients showed significantly elevated lactic, adipic, glutaric, phosphoric, aconitic, palmitic, 2-hydroxyisobutyric, pyroglutamic, phthalic, azelaic, and glycolic acids relative to controls, with reduced 4-hydroxyphenyllactic acid and trends toward reduction of 3-(3-hydroxyphenyl)propionic and 3-hydroxyphenylacetic acids; CD patients showed elevated 2-hydroxyisovaleric acid. Lactic, glutaric, and palmitic acids were associated with active UC, while glycolic, phosphoric, and adipic acids characterized remission. Pathway analyses indicated alterations in energy and amino acid metabolism. Exploratory multivariate ROC analyses yielded area under the Curve (AUC) values of 0.754 (IBD vs. controls), 0.904 (UC vs. controls), 0.798 (CD vs. controls), and 0.764 (UC vs. CD). These findings identify candidate urinary organic acid biomarkers associated with IBD status and UC disease activity, warranting validation in larger, independent, longitudinal cohorts before non-invasive tools for disease stratification and personalized management of UC and CD can be developed.

## 1. Introduction

Inflammatory bowel disease (IBD) prevalence has increased by approximately 47% globally between 1990 and 2019 [1]. During the same period, Argentina experienced a particularly striking escalation in IBD incidence, rising from 2 to 70 cases per 100,000 individuals [2], mirroring the epidemiological transition observed in industrialized nations [3]. IBD comprises two chronic inflammatory disorders, ulcerative colitis (UC) and Crohn’s disease (CD), arising from aberrant immune responses to the gut microbiota in the context of environmental triggers and genetic susceptibility [4,5]. Despite this growing burden, current diagnostic approaches remain suboptimal: clinical biomarkers lack adequate sensitivity and specificity for early detection, while definitive diagnosis requires invasive endoscopic, radiological, and histopathological evaluation [6]. Earlier identification of IBD could significantly reduce the burden of advanced intestinal inflammation and improve patient outcomes [7].

In IBD, gut microbiota homeostasis is disrupted in a state termed dysbiosis, characterized by reduced microbial diversity, loss of protective commensal populations, and expansion of potentially colitogenic pathobionts [8]. This dysbiotic state drives downstream metabolic consequences that metabolomic profiling is uniquely suited to capture, as individual metabolic signatures integrate genetic predisposition, lifestyle, diet, and gut microbiota activity, the latter contributing to over 69% of blood-detectable metabolites [8,9]. In patients with IBD, characteristic disruptions include reductions in short-chain fatty acids, secondary bile acids, and indole derivatives alongside elevations in sphingolipids and amino acid catabolites [10]. These metabolites modulate intestinal immune homeostasis, epithelial barrier integrity, and inflammatory signaling, and their altered abundance has been associated with the inflammatory environment characteristic of IBD [10]. Alterations in amino acid and energy metabolism further reflect adaptive responses to inflammation and nutritional stress, positioning metabolite profiling as a powerful tool for disease monitoring [11,12].

The biological matrix selected critically shapes the information obtained from metabolomic analyses. While serum metabolomics offers a window into systemic alterations and fecal metabolomics provides insights into intestinal microbial metabolism, both approaches have practical limitations for frequent monitoring [13,14]. Urine provides several advantages: non-invasive collection, sample abundance and stability, and the capacity to integrate both dietary and systemic microbial metabolic activity [15]. Recent studies have further supported this approach, demonstrating that targeted urinary metabolomic panels focused on central carbon metabolism can accurately differentiate UC and CD from controls using machine learning algorithms [16]. Nevertheless, urinary metabolomic profiles in IBD remain understudied, particularly in South American populations, which may harbor distinct metabolic signatures shaped by regional genetic, dietary, and environmental factors.

To address this gap, we conducted urinary organic acid profiling by GC-MS in an adult South American IBD cohort with the aim of identifying candidate biomarkers for non-invasive disease monitoring. We show that urinary organic acid signatures distinguish IBD patients from healthy controls, differentiate UC from CD, and could reflect disease activity states, with pathway analyses revealing coordinated alterations in energy and amino acid metabolism, collectively supporting urinary organic acid profiling as a candidate non-invasive tool for IBD diagnosis and monitoring.

## 2. Results

### 2.1. Clinical Characteristics of the South American Patient Cohort

This study included urine samples from 30 participants: 10 healthy controls, 10 UC patients, and 10 CD patients (Table 1). Groups were demographically comparable in age (median 34.5–39.5 years, one-way ANOVA, *p* = 0.488) and sex distribution (chi-square test, *p* = 0.999). Disease activity differed between subtypes: 80% of UC patients had active disease by Mayo Clinic Score (MCS), while 60% of CD patients were in remission by Harvey–Bradshaw Index (HBI). All UC patients received mesalazine; 30% also received corticosteroids. Among CD patients, 60% received either azathioprine or mesalazine. CD patients more frequently received biological therapies than UC patients (70% vs. 20%, respectively). Regarding disease distribution, half of UC patients had left-sided colitis, and 40% had proctitis, while 60% of CD patients presented with perianal disease.

### 2.2. Urinary Metabolomic Profiling Reveals Distinct Patterns in IBD Patients

**Figure 1:**
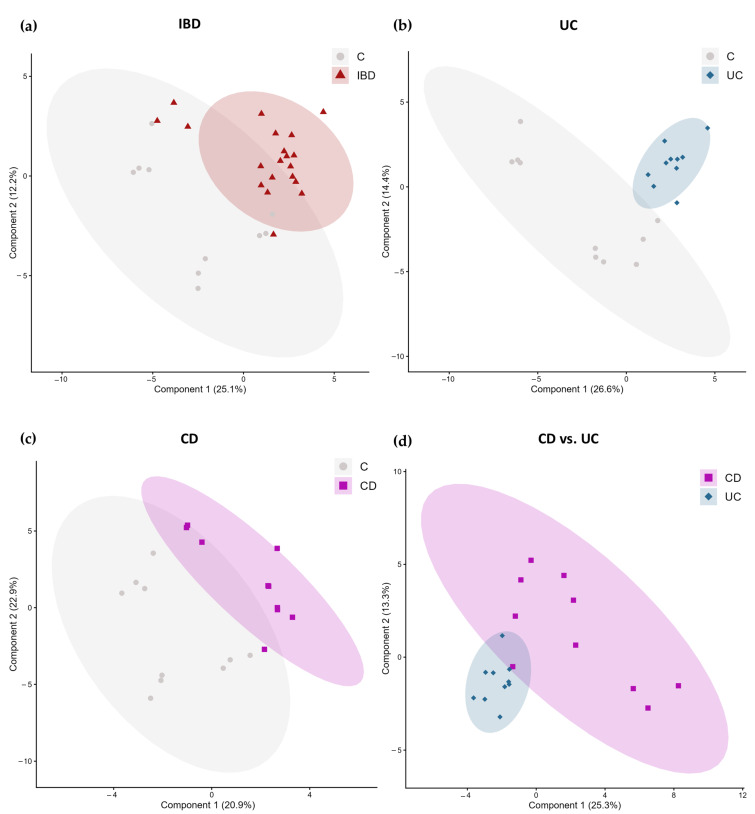
Distinct urinary metabolomic profiles are detected in patients with IBD. PLS-DA score plots showing the first two components with explained variances in parentheses. Each point represents an individual sample: gray circles for C, blue diamonds for UC, and purple squares for CD. Ellipses represent 95% confidence regions. The comparisons were performed between the following groups: IBD vs. C (**a**), UC vs. C (**b**), CD vs. C (**c**), and CD vs. UC (**d**). PLS-DA score plots are presented for visualization purposes only and should not be interpreted as evidence of robust group separation in the absence of statistically significant permutation testing.

Supervised Partial Least Squares Discriminant Analysis (PLS-DA) suggested clear separation between IBD patients and C (Figure 1a), UC and C (Figure 1b), CD and C (Figure 1c), and CD versus UC (Figure 1d). Unsupervised hierarchical clustering of the top 25 differential metabolites provided additional context for these metabolic differences, revealing broad urinary profile distinctions across groups, particularly for UC (Figure S2).

### 2.3. Distinct Metabolite Concentrations Discriminate Between Healthy Controls and IBD Subtypes

**Figure 2:**
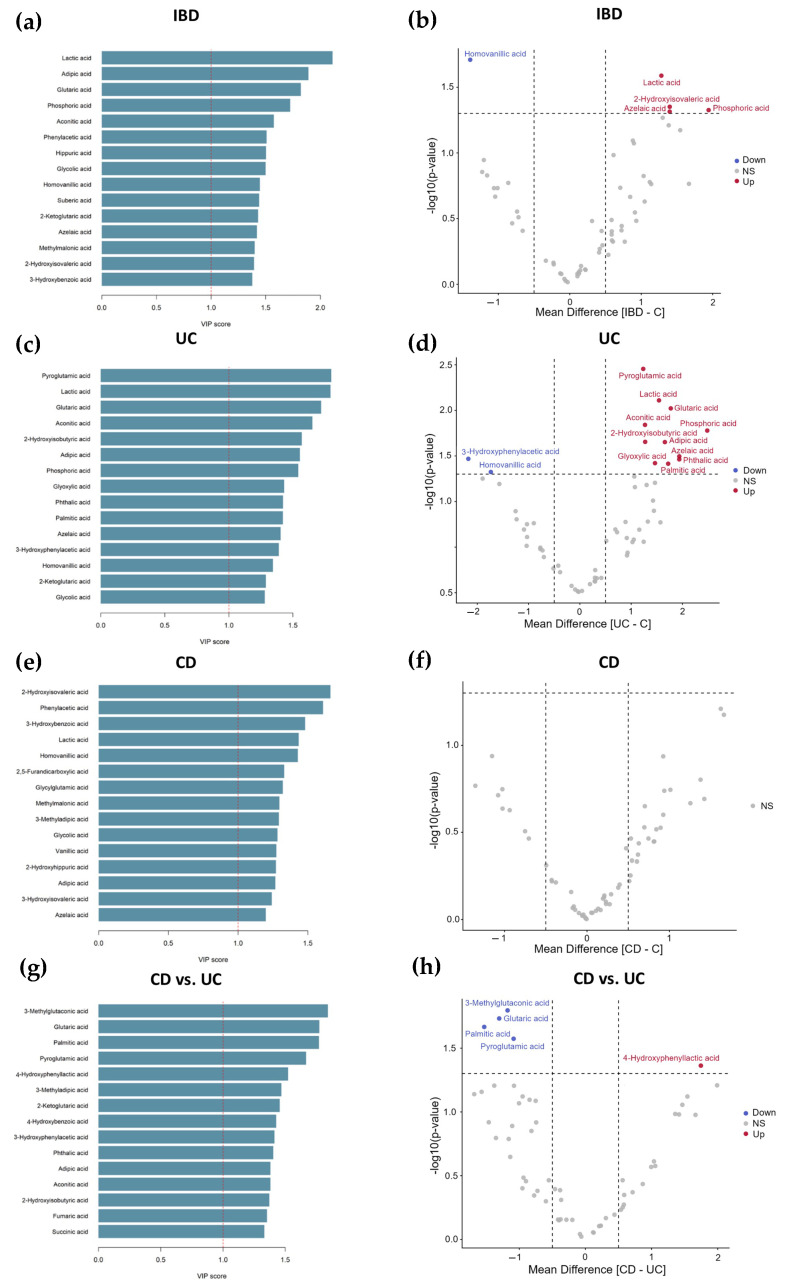
Discriminatory urinary metabolites distinguish patients with IBD from healthy controls. VIP scores (**a**,**c**,**e**,**g**) display the top 15 metabolites with VIP > 1 (red dashed lines) identified by PLS-DA from the component with the highest explained variance. Volcano plots (**b**,**d**,**f**,**h**) were generated using MetaboAnalyst. Threshold was set to 1.5 for the mean difference (mean difference, *x*-axis, vertical grey dashed lines set at 0.5) and 0.1 for the *t*-test *p*-value (*y*-axis, horizontal grey dashed lines). Both analyses compare: IBD vs. C (**a**,**b**), UC vs. C (**c**,**d**), CD vs. C (**e**,**f**), and CD vs. UC (**g**,**h**).

PLS-DA identified metabolites with Variable Importance in Projection (VIP) scores >1.0 as significant contributors to group separation. For IBD versus C, primary discriminatory metabolites included lactic, azelaic, phosphoric, and 2-hydroxyisovaleric acids, all elevated in IBD patients, while homovanillic acid was reduced (Figure 2a,b). For UC versus C, the most distinctive metabolites included pyroglutamic, lactic, glutaric, aconitic, phosphoric, 2-hydroxyisobutyric, adipic, azelaic, glyoxylic, phthalic, and palmitic acids, all elevated in UC patients, alongside reduced 3-hydroxyphenylacetic and homovanillic acids in UC patients (Figure 2c,d). For CD versus C, 2-hydroxyisovaleric and phenylacetic acids showed the strongest discriminatory contribution by VIP score (Figure 2e), though no metabolite reached statistical significance in direct comparisons (Figure 2f). The UC versus CD comparison identified reduced 3-methylglutaconic, glutaric, palmitic, and pyroglutamic acids alongside increased 4-hydroxyphenyllactic acid in CD relative to UC patients (Figure 2g,h).

When model validation was carried out using leave-one-out cross-validation (LOO-CV) (Table S1), the UC vs. C comparison showed the highest discriminatory accuracy (75%), with a statistically significant permutation *p*-value (*p* = 0.038); the corresponding model showed high sensitivity for UC (80%; 95% CI: 44.4–97.5%) and moderate specificity (70%; 95% CI: 34.8–93.3%). The C vs. IBD and CD vs. UC comparisons reached 70% accuracy (*p* = 0.166 and *p* = 0.084, respectively). C vs. IBD showed high sensitivity for IBD (85%; 95% CI: 62.1–96.8%) but low specificity (40%; 95% CI: 12.2–73.8%), while CD vs. UC showed the opposite pattern, with higher specificity for UC (80%; 95% CI: 44.4–97.5%) than sensitivity for CD (60%; 95% CI: 26.2–87.8%). Notably, despite reaching 70% accuracy, cross-validated Q^2^ values for the C vs. IBD and CD vs. UC models were low (Q^2^ = 0.0114 and 0.0105, respectively), indicating limited generalizable predictive power beyond the observed sample, consistent with the borderline/non-significant permutation *p*-values. The C vs. CD comparison showed the lowest accuracy (55%, *p* = 0.462), with sensitivity and specificity close to chance level (50% and 60%, respectively) and wide, overlapping confidence intervals. Overall, these results suggest that metabolomic profiles allow partial discrimination between groups, with the strongest and most balanced separation observed between healthy controls and UC patients, while the remaining comparisons showed asymmetric sensitivity/specificity trade-offs and wider confidence intervals consistent with the limited sample size and the exploratory nature of these comparisons.

**Figure 3:**
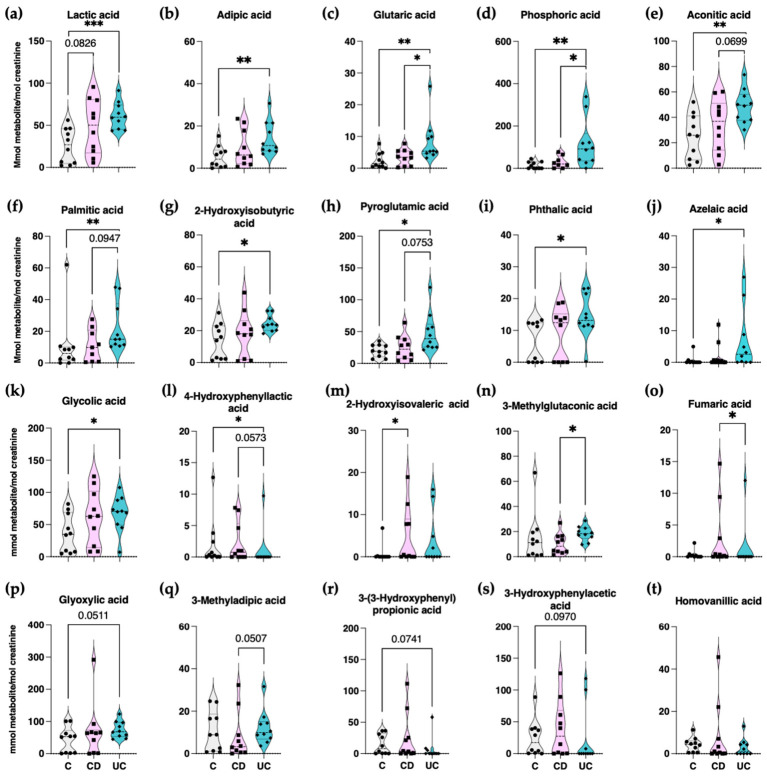
Urinary metabolites discriminate patients with IBD from healthy controls. Metabolites with VIP scores > 1 identified by PLS-DA from the following comparisons: IBD vs. C, UC vs. C, CD vs. C, and CD vs. UC are shown for the C, UC, and CD groups. Concentration in mmol of metabolites per mol of creatinine is shown for lactic (**a**), adipic (**b**), glutaric (**c**), phosphoric (**d**), aconitic (**e**), palmitic (**f**), 2-hydroxyisobutyric (**g**), pyroglutamic (**h**), phtalic (**i**), azelaic (**j**), glycolic (**k**), 4-hydroxyphenyllactic (**l**), 2-hydroxyisovaleric (**m**), 3-methylglutaconic (**n**), fumaric (**o**), glycoxylic (**p**), 3-methyladipic (**q**), 3-(3-hydroxypheyl)propionic (**r**), 3-hydroxyphenylacetic (**s**) and homovanillic (**t**) acid. Data normality was assessed using the Shapiro–Wilk test. For normally distributed data, Student’s *t*-test was applied; for non-normally distributed data, Mann–Whitney U test was used (* *p* < 0.05, ** *p* < 0.01, *** *p* < 0.001). *p*-values shown are unadjusted nominal values; no correction for multiple testing was applied.

Direct statistical comparisons of the most contributory metabolites (VIP > 1) confirmed these patterns (Table 2 and Table S3, Figure 3). In UC patients, lactic, adipic, glutaric, pyroglutamic, palmitic, phosphoric, 2-hydroxyisobutyric, phthalic, azelaic, glycolic, and aconitic acids were significantly elevated relative to controls, while 4-hydroxyphenyllactic acid was reduced compared to controls, with trends toward elevation of glyoxylic acid (*p* = 0.051) and toward reduction of 3-(3-hydroxyphenyl)propionic acid (*p* = 0.07) and 3-hydroxyphenylacetic acid (*p* = 0.09). Notably, glutaric, pyroglutamic, and palmitic acids were also elevated in UC versus CD patients. CD patients showed elevated 2-hydroxyisovaleric acid versus controls, and reduced glutaric, palmitic, and 3-methylglutaconic acids alongside elevated 4-hydroxyphenyllactic acid (*p* = 0.057) versus UC patients. No significant differences were observed for 3-hydroxyphenylacetic or homovanillic acid in this comparison. Given the predominance of active disease in UC and remission in CD, and the differences in treatment exposure between groups, these UC vs. CD differences should be interpreted with caution and cannot be attributed exclusively to disease subtype.

### 2.4. Disease Activity and Anatomical Location Associated with Specific Metabolic Signatures

Across the entire IBD cohort, lactic and 2-hydroxyisovaleric acids were significantly increased in remission, with glycolic acid showing a trend toward elevation at this disease state (Figure S3a,b,d). On the other hand, glutaric acid was significantly higher during active disease (Figure S3c). In CD, fumaric acid was significantly higher during active disease versus remission and controls (Figure S3e). In UC patients stratified by disease activity, lactic, glutaric, and palmitic acids were elevated in the active state relative to controls (Figure S4a,b,d), whereas glycolic, phosphoric, and adipic acids were higher specifically in remission (Figure S4c,e,f).

Regarding anatomical distribution, suberic acid differed significantly, while aconitic and adipic acids showed a trend between UC proctitis and left-sided colitis (Figure S5a–c). On the other hand, CD patients showed elevated homovanillic acid in perianal disease (Figure S5d). Given that subgroup sizes range from two to eight participants per activity or anatomical category, these analyses are strictly descriptive and exploratory.

### 2.5. Pathway Enrichment Analysis Reveals Distinct Metabolic Alterations in IBD

**Figure 4:**
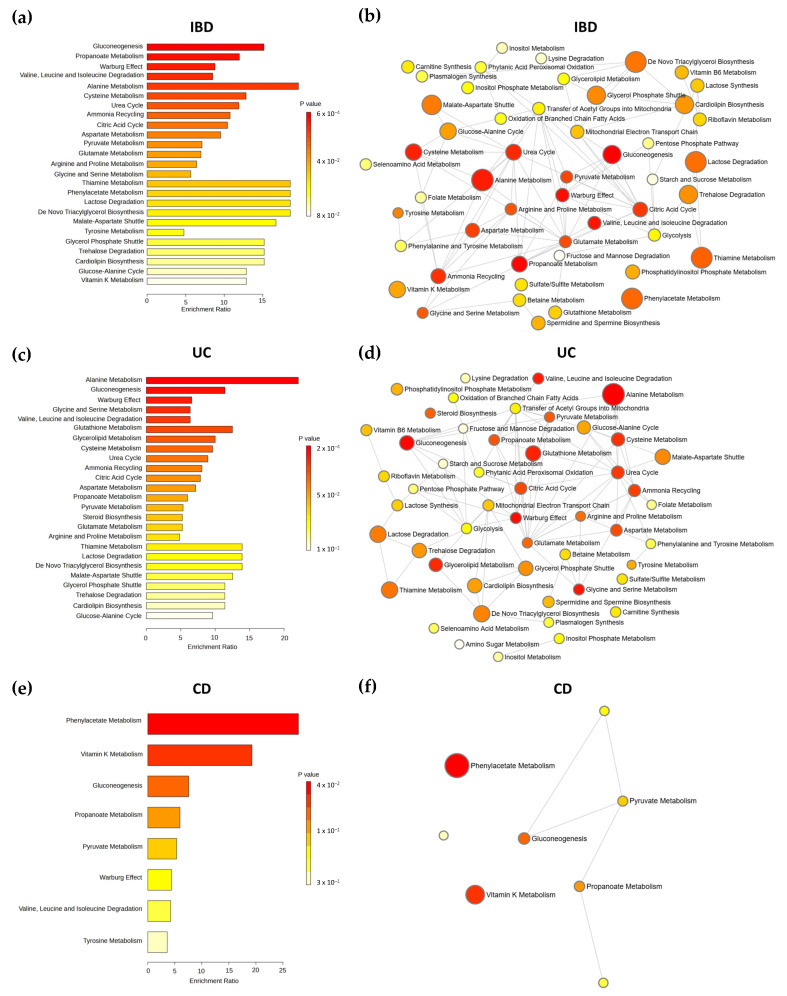
Altered metabolic pathways in patients with IBD. Metabolite pathway enrichment analysis was performed using ORA on the 15 metabolites with VIP scores >1 identified by PLS-DA, based on the Small Molecule Pathway Database (SMPDB). ORA results are shown for IBD (**a**), UC (**c**), and CD (**e**), while metabolic pathway mapping is presented for IBD (**b**), UC (**d**), and CD (**f**). Subfigures (**a**,**c**,**e**) display the enrichment overview, where horizontal bars represent different eukaryotic pathways and bar length indicates the enrichment ratio (*x*-axis). Color intensity corresponds to significance, with darker colors representing lower *p*-values. Subfigures (**b**,**d**,**f**) show metabolic pathway mapping, where circles represent individual pathways and circle size is proportional to the degree of enrichment (larger circles indicate higher enrichment). Color intensity corresponds to statistical significance, with darker shading indicating lower *p*-values. The comparative analyses included the following group comparisons: IBD vs. C, UC vs. C, and CD vs. C.

Over Representation Analysis (ORA) identified significant pathway enrichment across all comparisons, with pathways consistently ranked according to statistical significance. In the comparison of IBD versus healthy controls, the top-ranked pathways included gluconeogenesis, propanoate metabolism, the Warburg effect, valine, leucine, and isoleucine degradation, alanine metabolism, and cysteine metabolism, primarily involving energy and amino acid metabolism (Figure 4a). Metabolite set enrichment analysis further supported these metabolic changes (Figure 4b). In UC versus C, the top-ranked pathways included alanine metabolism, gluconeogenesis, the Warburg effect, glycine and serine metabolism, valine, leucine and isoleucine degradation, and glutathione metabolism, mainly involving amino acid and energy metabolism (Figure 4c,d). In CD versus C, phenylacetate metabolism, vitamin K metabolism, gluconeogenesis, propanoate metabolism, pyruvate metabolism, and the Warburg effect were among the top-ranked pathways, mainly involving energy- and microbiota-associated metabolic processes (Figure 4e,f).

### 2.6. Urinary Metabolites Suggest Biomarker Potential for IBD Diagnosis and Subtype Differentiation

**Figure 5:**
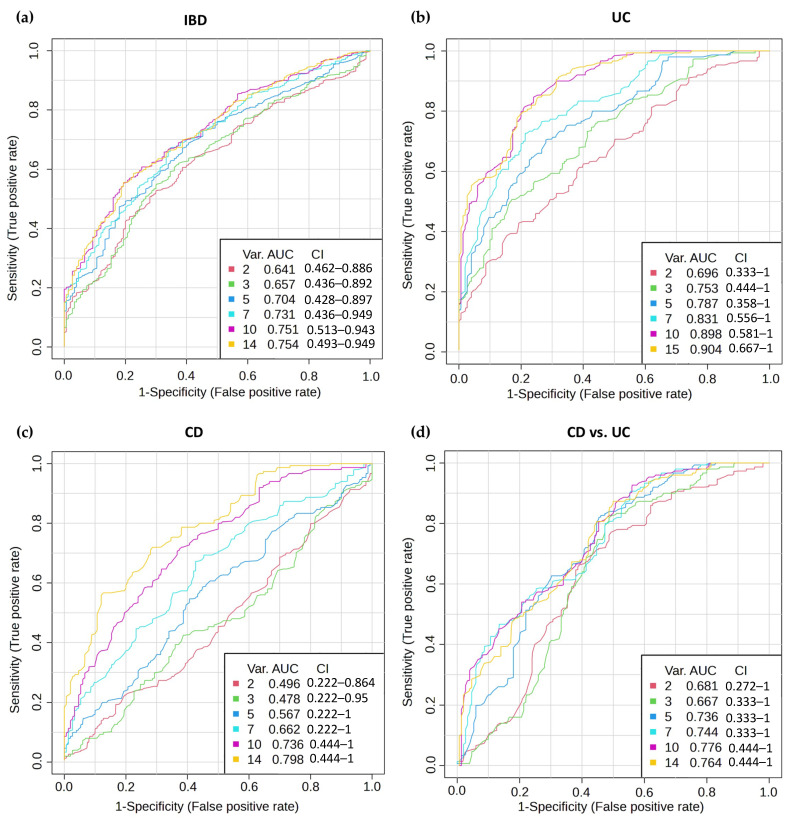
Urinary metabolites as potential biomarkers for IBD. ROC curves represent the collective performance of all biomarker models selected by PLS-DA, averaged across Monte Carlo cross-validation (MCCV) iterations. Comparative analyses included the following groups: IBD vs. C (**a**), UC vs. C (**b**), CD vs. C (**c**), and CD vs. UC (**d**).

Multivariate ROC analyses yielded optimal AUC values of 0.754 (14-metabolite model) and 0.641 (2-metabolite model) for IBD versus controls (Figure 5a and Figure S6a); 0.904 (15-metabolite model) and 0.696 (2-metabolite model) for UC versus controls (Figure 5b and Figure S6b); and 0.798 (14-metabolite model) for CD versus controls (Figure 5c and Figure S6c). Differentiation between UC and CD was more challenging, with an AUC of 0.764 for the optimal 14-metabolite model (Figure 5d and Figure S6d). Given the small sample sizes (*n* = 10 per group), these AUC values represent internal performance estimates subject to optimism bias and should not be interpreted as evidence of diagnostic utility. External validation in an independent cohort is required before any clinical inference can be drawn.

## 3. Discussion

This pilot study provides preliminary evidence of distinct urinary organic acid profiles obtained by GC-MS in adult IBD patients and their disease subtypes within a South American population, extending the growing body of evidence supporting urinary metabolomics as a viable non-invasive exploratory diagnostic strategy. The observed alterations in organic acid profiles are consistent with metabolic dysregulation, altered microbiota composition, or both. Coordinated changes in amino acid and energy metabolism pathways align with disease-associated metabolic reprogramming, while reductions in phenolic derivatives suggest a dysbiotic profile linked to impaired production of anti-inflammatory and antioxidant compounds. In this exploratory cohort, urinary metabolic signatures showed preliminary discriminatory performance between healthy controls and IBD patients, particularly for UC patients relative to healthy controls, and between UC and CD. Specific metabolites were further associated with disease activity and anatomical involvement, together suggesting that urine-based organic acid profiling may offer a practical complement to existing invasive diagnostic approaches.

Previous metabolomic studies have distinguished patients with IBD using various analytical platforms and biological matrices, predominantly serum, fecal, or tissue samples, reporting shifts in amino acid catabolism, bile acid dysregulation, and microbiota-derived metabolites [17,18,19,20]. Nuclear Magnetic Resonance (NMR)- and liquid chromatography (LC)-MS-based studies consistently described reduced short-chain fatty acids alongside increased ketone bodies and intermediates of the Krebs cycle [18,19], while GC-MS analyses in pediatric IBD cohorts revealed marked disruptions in energy metabolism and amino acid utilization [20,21]. Despite these advances, urine has remained comparatively underexplored in IBD, particularly in South American populations. Unlike serum- or fecal-based analyses, which primarily reflect systemic or luminal inflammation respectively, urinary metabolomics integrates host and microbial metabolic activity through a completely non-invasive sample [21,22,23]. Recent work in a Chinese cohort employing targeted Ultra High-Performance Liquid Chromatography (UHPLC)-MS/MS quantification of 49 central carbon metabolism metabolites identified xylose, L-fucose, GlcNAc, and citric acid as key discriminatory features, with diagnostic models achieving AUC values of 0.84 for UC and 0.93 for CD [16]. Notably, glycolic acid—identified as a discriminatory metabolite for UC in that targeted approach—also emerged as a significant marker in our semi-targeted GC-MS organic acid profiling, providing independent cross-platform validation of this finding. While previous urinary IBD studies have focused on treatment monitoring [22], pregnancy [23] or targeted central carbon metabolism [16], the present study complements these reports by identifying a distinct diagnostic organic acid signature in adult patients from a South American cohort, suggesting novel markers not previously proposed in this clinical and geographic context.

### 3.1. Microbiota-Derived Metabolites and Dysbiosis Signatures

The metabolic alterations observed in IBD likely reflect not only compositional dysbiosis but also shifts in the functional output of the microbiome [24]. As microbial composition was not directly assessed, the interpretation of specific metabolite origins as microbial remains is putative. The patterns observed are, however, consistent with dysbiosis-associated signatures previously reported in IBD cohorts [25]. Phenolic metabolites of putative microbial origin were consistently reduced: UC patients showed lower 4-hydroxyphenyllactic acid compared with controls, and a trend toward reduction of 3-(3-hydroxyphenyl)propionic acid relative to controls. These metabolites are generated by colonic bacteria through reductive metabolism of dietary polyphenols [26] and may exert antioxidant and anti-inflammatory effects in the intestinal mucosa, as suggested by observations from cancer chemoprevention research [27]. Their depletion parallels reports of more pronounced loss of beneficial microbial functions in UC relative to CD [8,25], and may contribute to the heightened mucosal oxidative burden characteristic of active UC. Altered levels of 2-hydroxyisobutyric, 2-hydroxyisovaleric, adipic, azelaic, and phthalic acids may further reflect disruptions in microbial fermentation pathways [8,25]. Elevated lactic acid in UC may indicate impaired microbial cross-feeding, possibly related to depletion of lactate-utilizing, butyrate-producing taxa [28], though microbiome composition was not directly assessed. Reduced butyrate production has well-established consequences for colonocyte energy supply and epithelial barrier integrity [28]. The overall pattern of metabolite alterations observed here is consistent with such dysbiosis-associated changes, though causality cannot be established from this cross-sectional pilot study.

### 3.2. Host Metabolic Reprogramming in IBD

ORA revealed significant enrichment of amino acid metabolism pathways, including valine, leucine, and isoleucine degradation, and alanine, cysteine, and glutamate metabolism, alongside energy-related pathways including gluconeogenesis, propanoate metabolism, and the Warburg effect. The enrichment of the Warburg effect pathway across all IBD comparisons is consistent with a shift toward aerobic glycolysis, as previously reported in intestinal epithelial cells and activated immune cells during inflammation [29]. Elevated lactic acid regardless of disease activity status may suggest that some degree of metabolic dysregulation persists beyond clinical remission, though this interpretation requires confirmation in longitudinal studies with larger, activity-balanced cohorts. Additional pathway enrichment signals were observed for the urea cycle, ammonia recycling, and phenylalanine metabolism, suggesting potential perturbations in nitrogen handling and aromatic amino acid processing that warrant further experimental investigation [30].

UC patients exhibited elevated pyroglutamic and glutaric acids relative to both controls and CD patients, suggesting disturbances in glutathione turnover and altered host–microbiota metabolic interactions [31]. Enrichment of phenylalanine and tyrosine metabolism further suggests altered aromatic amino acid catabolism with potential implications for neurotransmitter production and epithelial barrier regulation [30]. Altered levels of aconitic and fumaric acids are consistent with disrupted tricarboxylic acid cycle (TCA) cycle intermediate availability; whether these reflect mitochondrial dysfunction or other aspects of cellular metabolism cannot be determined from these metabolite measurements alone. Elevated glycolic and glyoxylic acids may reflect the altered redox balance that accompanies mucosal oxidative stress in active disease. Lipid metabolism changes were moderate but subtype-specific, with elevated palmitic and azelaic acids in UC consistent with lipid peroxidation and oxidative stress [32].

### 3.3. Subtype-Specific Metabolic Signatures Differentiate UC from CD

Beyond the shared IBD signature, distinct metabolomic patterns were observed between CD and UC. However, because UC patients were predominantly in active disease and CD patients predominantly in remission, and because medication regimens differed substantially between groups, these differences may partly reflect disease activity and treatment effects rather than intrinsic pathophysiological distinctions. With this important caveat, subtype-specific metabolic features are discussed below as exploratory observations. CD patients showed a trend toward reduction of 3-methyladipic acid, a novel discriminatory marker identified in this study that has not been previously proposed as an IBD biomarker, suggesting subtype-specific differences in lipid metabolism. Fumaric acid, a TCA cycle intermediate with known immunomodulatory properties [33], was consistently higher in CD than UC patients and emerged as a potential marker of active disease in this subtype. However, whether elevated fumaric acid in CD reflects or contributes to the immunological environment characteristic of this subtype cannot be determined from our data. Pathway analyses revealed enrichment of phenylacetate metabolism, vitamin K metabolism, gluconeogenesis, and propanoate metabolism in CD, supporting the existence of metabolically distinct phenotypes between UC and CD that warrant further investigation.

### 3.4. Disease Activity and Anatomical Distribution Reflected in Urinary Metabolites

Urinary metabolites captured both disease activity and anatomical distribution, highlighting the potential for non-invasive monitoring. In UC, glutaric and palmitic acids were elevated exclusively during active disease, potentially reflecting inflammatory metabolism and mitochondrial stress [34], while glycolic, phosphoric, and adipic acids were higher during remission, suggesting that certain metabolic alterations persist beyond the resolution of clinical symptoms. Notably, aconitic acid exhibited context-dependent behavior: elevated in UC remission, while decreased in the active state. This apparent inconsistency likely reflects differences in the predominant cell populations contributing to urinary metabolites across different disease states: epithelial repair processes may dominate during UC remission, whereas immune cell activation prevails during active pan-IBD inflammation, each driving distinct metabolic outputs. Moreover, aconitic acid levels were found to be decreased in IBD patients because it is consumed by Immune-Responsive Gene 1 (IRG1) to produce itaconate, an anti-inflammatory metabolite, during an immune metabolic reprogramming state [35]. Lactic and glutaric acids remained elevated in both active and remission states, indicating sustained metabolic dysregulation that extends beyond active inflammation and may reflect ongoing subclinical mucosal injury or host–microbiota dysregulation [8,25]. In CD, fumaric acid emerged as a potential disease activity marker, with higher concentrations during active disease possibly reflecting mitochondrial dysfunction and altered gut microbial composition. These observations may hold clinical relevance by suggesting that metabolomic profiling could complement conventional inflammatory markers such as C-Reactive Protein (CRP) and fecal calprotectin in assessing true disease quiescence and identifying patients at risk of relapse, particularly in cases where endoscopic and biochemical remission do not fully align [8].

Anatomical disease distribution was also associated with distinct metabolic signatures: in UC, suberic acid differed significantly between proctitis and left-sided colitis, while aconitic and adipic acids showed trends toward difference between disease locations, potentially reflecting variation in inflamed mucosal surface area and regional microbial activity [25]. In CD, homovanillic acid, a terminal dopamine metabolite, showed distinct patterns in perianal disease. Taken together, these findings indicate that urinary metabolomics can capture disease subtype, activity, and anatomical distribution in a single non-invasive sample, consistent with emerging multi-omic models of IBD heterogeneity [8,25].

### 3.5. Limitations and Future Directions

Several important limitations merit consideration. The exploratory design, limited sample size of 30 participants, and cross-sectional nature preclude causal inference and limit generalizability. Therefore, findings should be regarded as hypothesis-generating rather than confirmatory.

Disease activity and treatment exposure differed substantially between UC and CD, reflecting the clinical profile of IBD patients typically seen in our region. The imbalance in disease activity (80% in active UC vs. 60% remission in CD), together with universal mesalazine use in UC and higher biologic exposure in CD (70% vs. 20%), represents a potential confounding factor that may partially explain some of the observed metabolomic differences between subtypes, as these medications are known to influence gut microbiota composition and host metabolism independently of disease activity. Because treatment exposure was unevenly distributed across disease subtypes and the biggest metabolomic differences were observed in UC rather than CD, treatment-specific effects could not be reliably separated from disease-associated metabolic signatures in this exploratory cohort. Subtype-specific findings should therefore be interpreted as hypothesis-generating, and independent validation in activity-balanced, medication-stratified cohorts is required before clinical conclusions can be drawn.

Dietary intake was recorded but not restricted; although no differences in total energy or macronutrient intake were detected between groups, specific dietary precursors of urinary organic acids (e.g., individual polyphenols or amino acid-rich foods) were not assessed, and residual dietary confounding cannot be excluded. Creatinine-based normalization, while supported by the absence of significant inter-group differences in creatinine excretion (one-way ANOVA, *p* = 0.1619), may still be influenced by muscle mass, sex, hydration status, and renal function; renal function was not explicitly measured, though participants with known renal impairment would have met exclusion criteria as a chronic inflammatory condition.

The absence of direct microbiome characterization limits the ability to attribute specific metabolite changes to microbial taxa, although the observed patterns are consistent with known dysbiosis signatures in IBD. Similarly, the absence of concurrent inflammatory biomarkers (CRP or fecal calprotectin) prevents comparison of urinary organic acid signatures with established diagnostic tools, limiting assessment of their incremental clinical value. Regional genetic, dietary, and environmental factors specific to this South American cohort may further influence metabolite profiles in ways that limit direct comparison with cohorts from other geographic regions.

On the analytical side, no multiple-testing correction was applied to univariate comparisons, increasing the risk of false-positive findings; reported significant differences should be regarded as hypothesis-generating. The high-dimensional multivariate models (14–15 metabolites) relative to the small group sizes raise the risk of overfitting, and the reported AUC values likely overestimate true diagnostic performance. Formal analytical validation—including intra- and inter-day precision, carry-over assessment, and quality control (QC)-based drift correction—was not performed in this study.

Future studies should incorporate formal analytical validation procedures, including intra- and inter-day precision assessment and quality control-based drift correction, as recently demonstrated in targeted urinary metabolomics approaches for IBD [16], alongside integrated microbiome profiling and longitudinal sampling to establish temporal stability of the identified candidate biomarkers.

Despite these limitations, our South American pilot study suggests that urinary metabolomic profiling captures distinct metabolic alterations associated with IBD, its major subtypes, disease activity, and anatomical involvement, likely reflecting coordinated changes in host and microbiota-related metabolism. The identification of organic acid signatures discriminating IBD from healthy controls (AUC 0.754) and UC from controls with strong discriminatory performance (AUC 0.904) supports urinary metabolomics as a promising non-invasive exploratory approach for disease stratification and monitoring. The moderate performance differentiating UC from CD (AUC 0.764) indicates that while subtype-specific signatures exist, robust clinical differentiation will require validation in larger, independent, longitudinal cohorts with balanced disease activity, standardized medication protocols, and integrated microbiome profiling. The convergence of findings across semi-targeted GC-MS profiling strengthens confidence in urinary metabolomics as a reproducible and platform-independent strategy for IBD biomarker discovery.

## 4. Materials and Methods

### 4.1. Study Population and Clinical Assessment

This cross-sectional clinical study recruited 30 participants (10 healthy controls, 10 UC patients, and 10 CD patients) through the Gastroenterology Department of the Hospital Privado Universitario de Córdoba (HPUC) (Figure S1). The total number of participants (*n* = 30) and the size of each group (*n* = 10) were based on feasibility considerations and comparable sample sizes in previous exploratory metabolomic studies of IBD. No formal power calculation was performed, given the exploratory nature of this investigation. To minimize selection bias, consecutive patients meeting eligibility criteria were enrolled. Healthy controls were recruited from the same geographic region to ensure comparability.

Inclusion criteria comprised participants older than 18 years: patients with IBD with clinical, radiological, and endoscopic diagnosis, and healthy controls without IBD history, other chronic inflammatory conditions or active digestive tract pathology diagnosed within the previous 6 months. Exclusion criteria included pregnancy, antibiotic use for more than 7 days within the 2 months prior to enrollment, abdominal surgery within the preceding 6 months, and neoplasia. IBD activity was assessed using the Mayo Clinic Score (MCS) for UC [36] or the Harvey–Bradshaw Index (HBI) for CD [37]. No serum CRP, fecal calprotectin or any other clinical biomarkers were systematically collected in this study.

### 4.2. Sample Collection and Processing

Urine samples were collected as first-morning void specimens. Participants were not asked to follow specific dietary restrictions prior to collection. Dietary intake and recent food consumption were systematically recorded. No differences in total energy or macronutrient were detected. Morning urine samples were processed within 24 h of collection, coded and stored at −80 °C in the Centro de Investigación en Medicina Traslacional “Severo R. Amuchástegui” (CIMETSA) of the Instituto Universitario de Ciencias Biomédicas de Córdoba (IUCBC). Sample processing and GC-MS analysis were performed by personnel blinded to clinical diagnosis and disease activity status. Urinary organic acid profiles of participants were measured as described before [38]. Briefly, creatinine levels were measured to normalize organic acids (OAs) excretion to urine volume and no statistically significant differences in creatinine excretion were observed between groups (one-way ANOVA, *p* = 0.1619). To reduce measurement bias, all samples were processed using standardized protocols, and sample volumes equivalent to 100 µg creatinine were processed with undecanedioic acid as an internal standard for normalization. Alpha-keto acids were oximated with hydroxylamine at 80 °C for 30 min. The pH was then adjusted to 14 using sodium hydroxide, followed by acidification to pH 2 with hydrochloric acid to favor short-chain organic acid extraction. A liquid–liquid extraction was performed twice using ethyl acetate; the organic phase was collected and evaporated under a nitrogen stream. Derivatization reaction to generate volatile trimethylsilyl derivatives was performed at 60 °C for 1 h by adding 50 µL of N,O-bis(trimethylsilyl)trifluoroacetamide containing trimethylchlorosilane (BSTFA + TMCS) and 50 µL of acetonitrile prior to injection.

### 4.3. Gas Chromatography-Mass Spectrometry

Samples were analyzed using a Clarus SQ 580 Network gas chromatograph system (Perkin Elmer, Waltham, MA, USA) with an Agilent J&W DB-5 column (30 m length, 0.25 mm internal diameter, 0.25 μm film thickness, Agilent, Santa Clara, CA, USA). Analysis parameters included split mode injection (1 µL volume, 1:25 split ratio), 275 °C injector temperature, 230 °C ion source, and 130 °C quadrupole. Temperature program: 70 °C initial with 5 min hold, 4.5 °C/min ramp to 300 °C final temperature, 61.0 min total run time. Ionization was performed in electron impact mode, recording mass spectra every 0.25 s over the range of 50–480 *m*/*z*.

### 4.4. Data Processing

Raw GC-MS data were processed using TotalChrom v. 6.3.1 (Perkin Elmer) and TurboMass v. 5.4.2 (Perkin Elmer) for noise removal, peak detection, isotope cluster identification, retention time alignment, and putative metabolite identification. Feature intensities were sum-normalized to parts per million (ppm) units within each sample and method, yielding a total of 62 OAs. OAs’ identification was based on retention index matching, spectral library comparison, and confirmation against individual authenticated standards. Formal analytical validation, including intra- and inter-day precision, carry-over assessment, and QC-based drift correction, was not performed in this study, which may limit the assessment of analytical reliability.

### 4.5. Metabolomic and Statistical Analysis

Metabolomic data analysis was performed using R (v. 4.5.2) and MetaboAnalyst 6.0. Metabolite concentrations were expressed as mmol of metabolite per mol of creatinine. Raw data underwent log-base-2 transformation followed by Pareto scaling for all analyses. Missing values were imputed with half the minimum positive value per variable, and zero values were replaced with half the minimum positive global value. No samples were excluded due to missing metabolite data. Hierarchical clustering of the top 25 differential metabolites was performed using Ward’s method with Euclidean distance. All analyses were performed for the following pairwise comparisons: IBD versus controls (C), UC versus C, CD versus C, and CD versus UC. PLS-DA models were built for each pairwise comparison (C vs. IBD, C vs. UC, C vs. CD, C vs. IBD, and CD vs. UC) using the mixOmics package (v. 6.34.0, Bioconductor 3.22) in R. The optimal number of latent components (1–5) was determined by LOO-CV using the maximum distance (max.dist) classification rule. Component selection was based on cross-validated predictive performance (Q^2^), applying a parsimony criterion to retain the smallest model with near-maximal predictive ability. When no component achieved Q^2^ > 0, a one-component model was retained. For the C vs. UC comparison, one component was selected a priori because additional components did not improve classification performance. R^2^X was calculated as the cumulative proportion of explained X-variance. Model significance was assessed by permutation testing (999 permutations). Sensitivity and specificity were calculated from leave-one-out cross-validated predictions, and exact 95% confidence intervals for sensitivity and specificity were estimated using the Clopper–Pearson method. The positive class was predefined for each comparison. Univariate comparisons shown in Figure 3 and Figure S5 and Table 2 represent nominal significance thresholds (*p* < 0.05) without correction for multiple testing, given the exploratory nature of this study. Pairwise comparisons shown in Figures S3 and S4 use correction for multiple testing by applying the Kruskal–Wallis test followed by Dunn’s post hoc test. Metabolites with VIP scores > 1 were considered important discriminatory features, and their normality was assessed using the Shapiro–Wilk test. Model validation was carried out using LOO-CV, and statistical robustness was evaluated through permutation testing with 100 iterations. All models converged at a single optimal component; full model performance metrics are detailed in Table S1. Volcano plots were constructed in R combining mean differences between groups (threshold absolute mean difference ≥ 0.5) and statistical significance (unpaired Student’s *t*-test, *p* < 0.05) without correction for multiple testing, given the exploratory nature of this study. Volcano plots used mean difference between group log_2_-transformed values on the *x*-axis (threshold |mean difference| ≥ 0.5) rather than fold change, given that log_2_-transformed and Pareto-scaled data do not directly yield fold change values. This threshold was selected to balance sensitivity and specificity in an exploratory context. Pathway enrichment analysis was conducted in MetaboAnalyst using ORA against the SMPDB, on the top VIP-selected metabolites from each PLS-DA model. This approach may be subject to instability given the small input metabolite set, and results should be interpreted as preliminary pathway hypotheses rather than confirmed enrichment findings. FDR-adjusted pathway *p*-values (adjusted *p* < 0.05) were used as the significance threshold. Candidate biomarker potential was independently evaluated using the ROC curve analysis in MetaboAnalyst. Multivariate ROC analysis employed PLS-DA with Monte Carlo cross-validation, while univariate analysis used bootstrap-estimated confidence intervals with Youden’s index for optimal cutoff determination; classification performance metrics are detailed in Table S2. Comparisons between two groups used Student’s *t*-test or Mann–Whitney U test as appropriate. Multiple group comparisons employed one-way ANOVA with Tukey’s post hoc test or Kruskal–Wallis with Dunn’s test for non-normally distributed data. Age differences were analyzed using one-way ANOVA, and sex distributions using a chi-square test. Statistical significance was set at *p* < 0.05. Concentration graphs were generated using GraphPad Prism (v9.2.0).

### 4.6. Data Availability

Individual participant data that underlie the results reported in this article, some of them shown in Table S3, after de-identification, are available from the corresponding authors upon reasonable request.

### 4.7. AI Language Model Assistance Disclosure

Claude Sonnet 4.6, developed by Anthropic (San Francisco, CA, USA), assisted in refining the written content of this manuscript. Claude Sonnet 4.6 provided suggestions and corrections based on the input provided by the user, enhancing the clarity and grammar of the text. Claude Sonnet 4.6 output was carefully revised by the user to ensure the right message was delivered. All mechanistic statements, statistical interpretations, and scientific conclusions were verified by the authors against the primary data and the peer-reviewed literature. No AI-generated content was included without author review and approval.

## 5. Conclusions

This pilot study identifies exploratory urinary organic acid signatures revealed by GC-MS associated with IBD status, UC disease activity, and anatomical distribution in a South American cohort. Elevated lactic, glutaric, palmitic, and related organic acids suggested coordinated alterations in energy and amino acid metabolism consistent with host metabolic reprogramming and dysbiosis-associated changes. Specific metabolites were further associated with disease activity states and anatomical distribution, suggesting urine-based profiling as a candidate, exploratory approach, warranting further investigation for non-invasive IBD monitoring. These findings are hypothesis-generating and require prospective validation in larger, independent, longitudinal cohorts with integrated microbiome profiling and standardized clinical characterization before any clinical inference can be drawn.

## Figures and Tables

**Table 1:** Clinical and demographic characteristics of study participants. A total of 30 individuals were included in three groups: healthy controls (C), *n* = 10; ulcerative colitis (UC), *n* = 10; and Crohn’s disease (CD), *n* = 10. Disease activity in patients with IBD was assessed clinically using MCS for UC and the HBI for CD.

	C (*n* = 10)	UC (*n* = 10)	CD (*n* = 10)	*p*-Value
**Age: median** **(25th, 75th percentile)**	37.5 (27.5, 44.25)	34.5 (26.3, 45.5)	39.5 (31.75, 52.25)	0.4888
**Sex** **(F/M)**	6/4 60%/40%	6/4 60%/40%	7/3 70%/30%	0.9996
**Disease activity**				
**Remission**	-	2 (20%)	6 (60%)	
**Mild Activity**	-	6 (60%)	2 (20%)	
**Moderate Activity**	-	2 (20%)	2 (20%)	
**IBD therapy**				
***Conventional***				
Azathioprine	-	3 (30%)	3 (30%)	
Mesalazine	-	10 (100%)	3 (30%)	
Corticosteroids	-	3 (30%)	0 (0%)	
***Biological***				
Infliximab	-	0 (0%)	1 (10%)	
Adalimumab/Humira	-	2 (20%)	5 (50%)	
Vedolizumab	-	-	1 (10%)	
**IBD location**				
***Ulcerative colitis***				
Proctitis	-	4 (40%)	-	
Left-sided	-	5 (50%)	-	
Extensive	-	1 (10%)	-	
***Crohn’s disease***				
Colonic	-	-	5 (50%)	
Ileocolonic	-	-	4 (40%)	
Ileum			1 (10%)	
No Perianal disease	-	-	4 (40%)	
Perianal disease	-	-	6 (60%)	

**Table 2:** Summary of discriminatory urinary metabolites identified by PLS-DA analysis. Differential urinary metabolites are summarized according to the direction of change across comparisons. VIP scores were obtained from PLS-DA, considering metabolites with VIP > 1 from the component with the highest explained variance.

Metabolite Name	UC vs. C	CD vs. C	CD vs. UC
Lactic acid	↑	ns	ns
Adipic acid	↑	ns	ns
Glutaric acid	↑	ns	↓
Phosphoric acid	↑	ns	↓
Aconitic acid	↑	ns	ns
Palmitic acid	↑	ns	ns
2-Hydroxyisobutyric acid	↑	ns	ns
Pyroglutamic acid	↑	ns	ns
Phthalic acid	↑	ns	ns
Azelaic acid	↑	ns	ns
Glycolic acid	↑	ns	ns
4-Hydroxyphenyllactic acid	↓	ns	ns
2-Hydroxyisovaleric acid	ns	↑	ns
3-Methylglutaconic acid	ns	ns	↓
Fumaric acid	ns	ns	↑
Glyoxylic acid	ns	ns	ns
3-Methyladipic acid	ns	ns	ns
3-(3-Hydroxyphenyl) propionic acid	ns	ns	ns
3-Hydroxyphenylacetic acid	ns	ns	ns
Homovanillic acid	ns	ns	ns

Arrows indicate significantly increased (↑) or decreased (↓) metabolite concentrations with a directionality relative to the first-named group (e.g., ↑ in CD vs. UC column means elevated in CD relative to UC); ns indicates non-significant differences. Comparative analyses included UC versus C, CD versus C, and CD versus UC. Significance in the comparison is obtained when *p* < 0.05. Data normality was assessed using the Shapiro–Wilk test. For normally distributed data, Student’s *t*-test was applied; for non-normally distributed data, Mann–Whitney U test was used (*p* < 0.05, *p* < 0.01, *p* < 0.001). No multiple-testing adjustment was applied due to the exploratory nature of our study.

## Data Availability

Individual participant data that underlie the results reported in this article, after de-identification, are available from the corresponding authors upon reasonable request.

## Supplementary Materials

The following supporting information can be downloaded at: https://www.mdpi.com/article/10.3390/ijms27146318/s1.

## Abbreviations

The following abbreviations are used in this manuscript:

AUC	Area under the Curve
BSTFA+ TMCS	N,Obis(trimethylsilyl)trifluoroacetamide trimethylchlorosilane
CD	Crohn’s Disease
CRP	C-Reactive Protein
FDR	False Discovery Rate
GC-MS	Gas Chromatography Mass Spectrometry
HBI	Harvey–Bradshaw Index
HPUC	Hospital Privado Universitario de Córdoba
IBD	Inflammatory Bowel Disease
IRG1	Immune-Responsive Gene 1
LOO-CV	Leave-One-Out Cross-Validation
LC-MS	Liquid Chromatography Mass Spectrometry
MCS	Mayo Clinic Score
MCCV	Monte Carlo Cross-Validation
MS	Mass Spectrometry
NMR	Nuclear Magnetic Resonance
OAs	Organic Acids
ORA	Over-Representation Analysis
PLS-DA	Partial Least Squares Discriminant Analysis
QC	Quality Control
ROC	Receiver Operating Characteristic
SMPDB	Small Molecule Pathway Database
TCA	Tricarboxylic Acid Cycle
UC	Ulcerative Colitis
UHPLC-MS	Ultra High-Performance Liquid Chromatography Mass Spectrometry
VIP	Variable Importance in Projection
