# Supplementary material for: Urinary Organic Acid Profiling by GC-MS Reveals Distinct Metabolic Signatures for Non-Invasive Diagnosis and Disease Activity Monitoring of Inflammatory Bowel Disease
Source: Int J Mol Sci. 2026 Jul 16;27(14):6318. doi: 10.3390/ijms27146318 (PMC13409919; doi:10.3390/ijms27146318)
Supplement: Supplementary file 1 [file ijms-27-06318-s001.zip › ijms-4411934-supplementary.pdf]

Article

Urinary Organic Acid Profiling by GC-MS Reveals Distinct Metabolic Signatures for Non-Invasive Diagnosis and Disease Activity Monitoring of Inflammatory Bowel Disease

Supplementary Material

Table S1: Performance metrics of PLS-DA models for pairwise group comparisons. For each comparison, leave-one-out cross-validation (LOO-CV) was performed to determine the optimal number of components and estimate classification error and accuracy. The optimal number of components obtained in all comparisons was 1. R^2X and Q^2 were calculated as measures of explained variance and predictive ability, respectively. Statistical significance was assessed by permutation testing (n = 999 permutations), with p-values indicating the proportion of permuted models achieving accuracy equal to or greater than the observed model. Sensitivity and specificity (with 95% Clopper-Pearson confidence intervals) were derived from the LOO-CV confusion matrix, using CD, UC, and IBD as the positive class in their respective comparisons against C, and CD as the positive class in the CD vs. UC comparison.

Comparison	Samples	Metabolites	Minimum Error	Accuracy	R^2X	Q^2	Permutation p-value	Sensitivity (95% CI)	Specificity (95% CI)
**IBD vs. C**	30	61	0.30	0.70	0.2532	0.0114	0.166	0.85 (0,6211-0,9679)	0.4 (0,1216-0,7376)
**UC vs. C**	20	60	0.25	0.75	0.2631	0.2137	0.038	0.8 (0,4439-0,9748)	0.7 (0,3475-0,9333)
**CD vs. C**	20	61	0.45	0.55	0.2130	-0.3402	0.462	0.5 (0,1871-0,8129)	0.6 (0,2624-0,8784)
**CD vs. UC**	20	60	0.30	0.70	0.2576	0.0105	0.084	0.6 (0,2624-0,8784)	0.8 (0,4439-0,9748)

Table S2. Cross-validation performance metrics for ROC classification models generated in MetaboAnalyst using VIP-selected metabolites. Confusion matrices obtained by cross-validation were used to calculate true negatives (TN), false positives (FP), false negatives (FN), true positives (TP), accuracy, sensitivity, and specificity for each comparison. The analyses were performed in MetaboAnalyst using the top 15 metabolites selected from PLS-DA VIP scores generated in R.

Comparison	True Negatives (TN)	False Positives (FP)	False Negatives (FN)	True Positives (TP)	Accuracy	Sensitivity	Specificity
**IBD vs. C**	7	6	3	14	0.70	0.82	0.54
**UC vs. C**	8	3	2	7	0.75	0.78	0.73
**CD vs. C**	9	3	1	7	0.80	0.88	0.75
**CD vs. UC**	6	4	4	6	0.60	0.60	0.60

Table S3. Individual-level features of the participant cohort and key metabolite concentrations. Age (**A**), sex (**S**), disease activity (**DA**, R-Remission, Al-Mild activity, and Am-Moderate activity), location (L, CD: Pa-Perianal, N-No perianal, UC: Pr-Proctitis, and Ls-left sided), treatment exposure (Y-Yes, N-No, A-azathioprine, M-mesalazine, C-corticosteroids and/or B-biologicals: N-None, I-infliximab, H-Humira, A-Adalimumab, and V-Vedolizumab), creatinine (Cr, mg/dL) and normalized values of metabolites (mmol/mol of creatinine) are displayed for each participant (**P**) of the study cohort. OAs: adip:adipic, glut:glutaric, phosp:phosphoric, acon:aconitic, palm: palmitic, 2-hbu:2-hydroxybutiric, pyrog:pyroglutamic, phta: pthalic, azel: azelaic, gly: glycolic, 4-hpl: 4-hydroxyphenillatic, 2-hpv: 2-hydroxyphenilvaleric, 3-mgl: 3-methylglutaconic, fum: fumaric, glyo: glyoxylic, 3-ma: 3-methyladipic, 3-3-h: 3-(3-Hydroxyphenyl)propionic, 3-hpa: 3-hydroxyphenylacetic, homo: homovanillic. m:missing value.

**P**	**A**	**S**	**DA**	**L**	**A**	**M**	**C**	**B**	**Cr**	**lactic**	**adip**	**glut**	**phosp**	**acon**	**palm**	**2-hbu**	**pyrog**	**phta**	**azel**	**glyc**	**4-hpl**	**2-hpv**	**3-mgl**	**fum**	**glyo**	**3-ma**	**3-3-h**	**3-hpa**	**homo**
**C1**	58	F	-	-	-	-	-	-	107	46,25	7,49	1,8	24,59	32,81	8,53	13,8	16,82	12,54	0	43,98	0	0	12,17	0	62,49	8,91	3,68	38,62	4,67
**C2**	39	F	-	-	-	-	-	-	58	32,73	7,95	0	12,3	25,29	0	16,12	19,74	12,15	0	33,68	0	0	10,24	0	51,57	24,72	36,36	40,49	4,1
**C3**	29	F	-	-	-	-	-	-	133	45,93	0	4,48	0	40,26	0	31,21	28,52	12,36	0	36,02	0	0	66,88	0	54,39	16,7	12,26	88,88	5,04
**C4**	28	F	-	-	-	-	-	-	262	21,24	15,27	7,74	31,66	43,86	10,54	19,81	35,28	11,25	0	73,71	0	0	13,8	0	101,62	16,47	36,22	0	11,29
**C5**	45	F	-	-	-	-	-	-	109	39,53	10,52	4,88	29,88	52,13	62,02	22,64	29,04	1,24	0	82,06	3,8	0	19,33	2,18	100,72	24,4	25,85	29,06	7,11
**C6**	41	F	-	-	-	-	-	-	51	56,24	6,48	2,58	45,06	26,48	9,9	24,42	27,28	13,29	0	67,39	2,4	6,82	21,75	0	53,79	8,81	31,01	36,77	5,66
**C7**	25	M	-	-	-	-	-	-	153	2,21	1,78	0,63	0	2,43	8,55	2,28	6,54	0	4,97	5,12	0,24	0	1,47	0,14	0,54	0,73	0	0,39	0,49
**C8**	27	M	-	-	-	-	-	-	262	2,53	0,52	0,41	0	7,05	3,51	1,23	11,62	0	0	4,84	0,73	0	1,12	0,19	1,84	0,73	0	4,45	0,65
**C9**	44	M	-	-	-	-	-	-	216	6,73	0,75	0,57	0	4,87	2,49	2,32	6,69	0	0,24	7,82	0,36	0,18	1,16	0,2	2,37	1,27	0,49	5,76	0,58
**C10**	36	M	-	-	-	-	-	-	100	5,35	2,11	1,05	0	13,86	2,35	3,07	18,43	0	0,7	9,56	12,65	0	2,41	0,43	2,71	2,58	0,52	1,18	2,88
**CD1**	45	F	Al	Pa	Y	Y	N	I	85	82,25	17,78	7,78	0	59,24	18,65	17,59	64,09	13,89	0	97,48	7,82	12,5	16,5	14,68	92,38	32,32	72,25	126,17	45,69
**CD2**	48	F	Al	N	N	N	N	N	79	41,41	6,78	3,33	25,03	41,85	5,57	17,29	14,14	18,51	0	63,35	0	0	11,67	9,43	63,67	5,87	3,6	40,01	0
**CD3**	34	F	R	N	N	N	N	H	217	79,8	4,86	4,77	65,44	44,86	27,63	20,85	37,63	14,11	6,35	124,93	0	18,91	13,3	0	63,7	8,72	21,19	14,85	3,31
**CD4**	46	M	R	Pa	Y	Y	N	A	128	63,98	21,74	5,57	16,33	31,94	m	24,07	28,43	0	0	114,57	7,44	7,8	16,22	0	291,81	0	25,43	60,91	7,1
**CD5**	65	F	R	Pa	N	N	N	N	80	58,89	9,78	3,64	77,22	60,22	11,74	43,93	29,53	18,79	0	62,11	0	7,75	26,96	0	65,78	23,55	111,38	89,21	22,07
**CD6**	65	M	Am	N	N	N	N	H	171	24,74	5,73	2,97	15,78	25,57	9,84	32,77	16,26	11,64	0	44,28	0	0	4,18	2,9	42,45	3,59	0	0	0
**CD7**	28	M	R	N	N	N	N	H	144	95,54	23,44	5,4	38,15	48,29	22,81	18,53	40,71	13,29	11,93	73,58	4,6	0	4,84	0	66,97	2,95	0	0	0
**CD8**	33	F	R	N	N	N	N	H	106	19,55	1,77	0,57	m	15,62	0,8	1,23	4,23	0	0,73	16,23	0,99	0,2	4,05	0,43	2,51	0,56	0	47,5	0,57
**CD9**	34	F	R	N	Y	N	N	N	213	5,68	2,26	0,55	m	10,12	0,9	2,23	4,93	0	0,68	8,27	1,01	0,49	3,05	0,34	1,48	1,59	4,41	0,46	1,14
**CD10**	20	F	Am	Pa	N	N	N	V	41	10,39	0,73	0,26	0	2,9	0,53	0,96	9,17	0	0,45	7,84	0,51	0	1,43	0,16	0,27	0,56	0,56	1,95	0,15

**UC1**	45	M	R	Pr	Y	Y	Y	N	240	64,25	21,45	5,44	338,57	62,36	17,77	22,36	75,62	11,36	21,24	87,79	0	14,32	15,83	0	55,22	14,37	0	0	0
**UC2**	24	F	Al	Pr	N	Y	N	N	269	58,9	8,88	9,91	118,62	73,5	11,54	24,1	26,86	21,5	0	69,92	0	0	20,2	0	84,13	9,18	0	0	4,33
**UC3**	32	M	Am	Ls	N	Y	Y	N	276	55,2	6,79	5,32	292,36	30,17	47,1	20,28	57,12	12,36	3,13	91,46	0	2,09	9,6	0	58,47	8,78	0	7,78	0
**UC4**	27	F	Al	Ls	N	Y	N	H	260	73,06	9,88	9,34	96,68	48,92	10,24	23,58	33,71	15,18	0	70,98	0	0	10,58	0	69,76	5,39	0	0	2,24
**UC5**	53	M	Al	Ls	N	Y	N	N	79	43,96	7,92	4,71	87,14	38,12	15,02	18,16	25,65	0,25	1,94	50,6	0	4,85	17,1	0	46,53	10,63	5,26	0	0
**UC6**	34	F	Al	Ls	N	Y	N	N	192	45,52	11,57	4,26	41,82	40,15	11,95	27,62	21,27	23,13	8,77	68,28	0	0	17,59	12,03	44,76	7,36	8,46	0	5,43
**UC7**	36	F	R	Pr	N	Y	N	N	91	60,33	30,72	5,18	122,2	50,92	10,73	23,64	54,46	13,94	0	107,62	9,73	15,97	23,8	0	96,54	31,62	58,06	100,38	0
**UC8**	22	F	Al	Pr	Y	Y	N	N	301	91,35	21,5	11,77	0	50,16	47,83	32,45	44	23,27	4,86	73,3	0	0	19,08	0	66,87	13,85	0	0	4,22
**UC9**	47	F	Al	Ls	N	Y	Y	N	126	77,86	17,05	25,81	26,78	56,71	34,24	32,42	119,66	11,81	0,27	7,06	0	0	22,72	0	123,16	17,21	0	118,01	12,98
**UC10**	35	M	Am	Ls	Y	Y	N	H	83	43,47	8,32	3,2	38,32	36,3	14,9	19,79	25,22	11,26	26,95	45,28	0	0	28,65	0	99,56	3,64	0	0	0

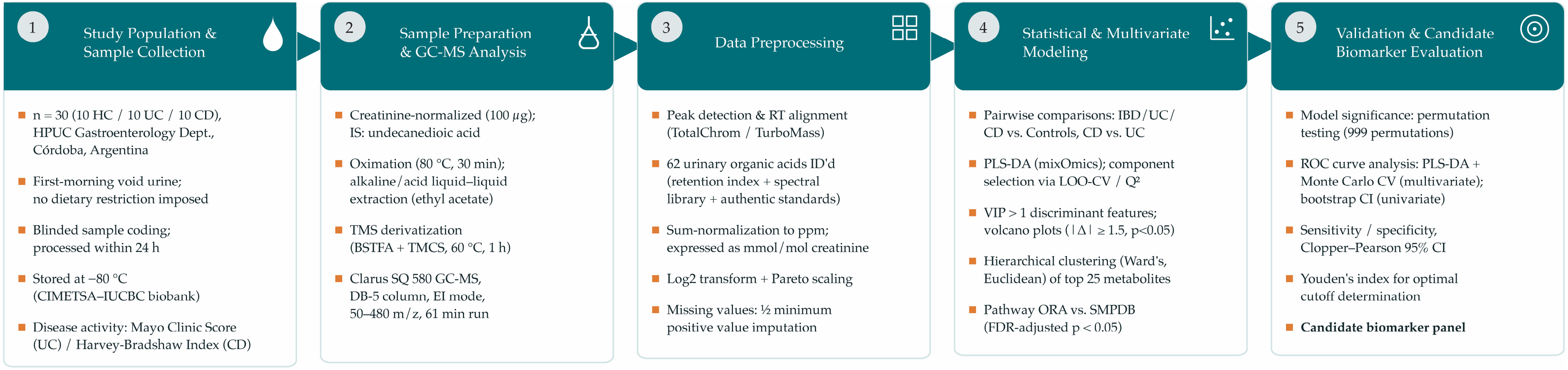

Figure S1: Analytical workflow for urinary organic acid profiling in IBD. Urine samples from 30 participants (10 controls, 10 UC, 10 CD) underwent GC-MS-based organic acid profiling following creatinine normalization and derivatization. Data preprocessing, PLS-DA and univariate statistical modeling, and ROC-based validation were used to identify candidate urinary biomarkers distinguishing IBD from controls, UC from CD, and reflecting disease activity. For complete description, please see Material and Methods section.

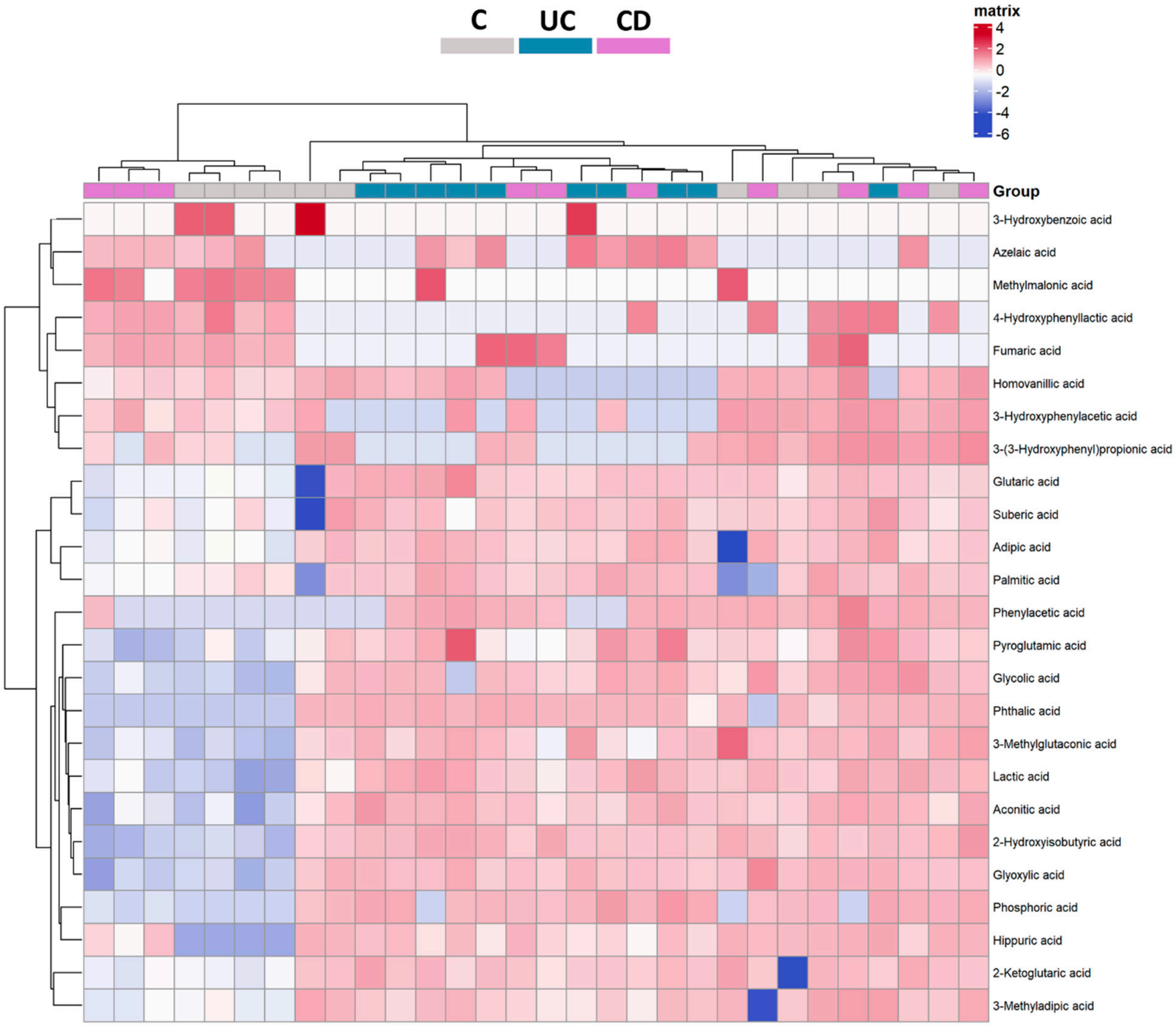

Figure S2: Urinary metabolomic profiling revealed distinct metabolic signatures that differentiate healthy controls (C) from patients with Inflammatory Bowel Disease (IBD)**.** A heatmap hierarchical clustering was generated for the top 25 differential metabolites using Euclidean distance and Ward's linkage algorithm (ward.D2). Metabolites were selected based on ANOVA p-values across the three groups. Each row represents a metabolite and each column an individual sample. Color intensity indicates standardized metabolite abundance (Z-score): blue for low, white for medium, and red for high concentrations. The top annotation bar shows group assignment: gray for healthy controls (C), blue for ulcerative colitis (UC), and purple for Crohn's disease (CD). Clusters represent the comparison between CD, UC and C groups.

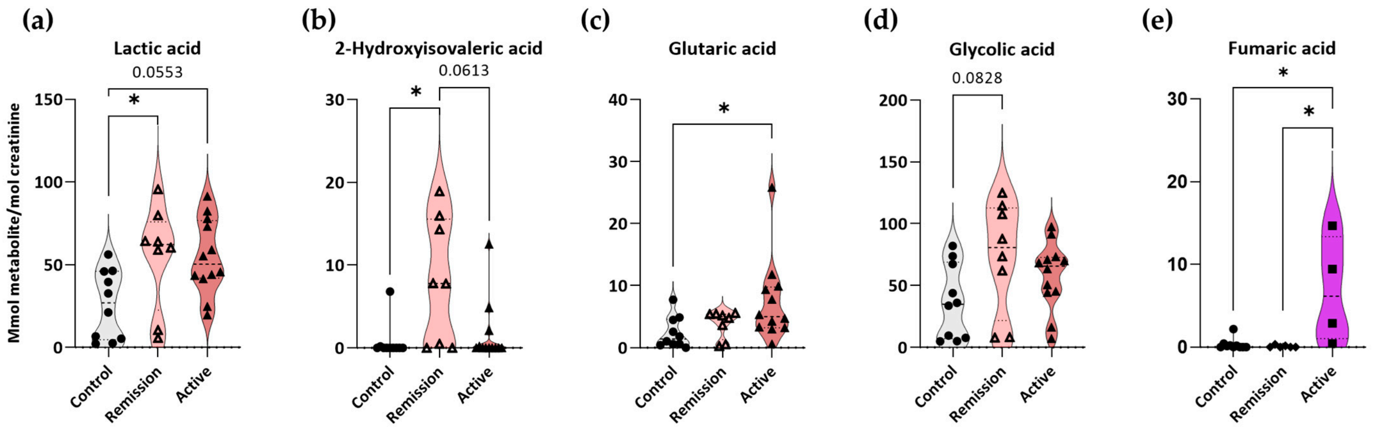

Figure S3: Specific metabolites associated with clinical disease activity in IBD and CD patients. Metabolites with VIP scores > 1 identified by PLS-DA comparing IBD vs. C (**a**-**d**) and CD vs. C (**e**) are shown stratified by clinical disease activity (remission, mild, moderate), as determined from endoscopic reports. Given the small sample size in some groups (n < 10), non-parametric analyses were applied directly. Therefore, group comparisons were performed using the Kruskal–Wallis test followed by Dunn's post-hoc test (*p<0.05).

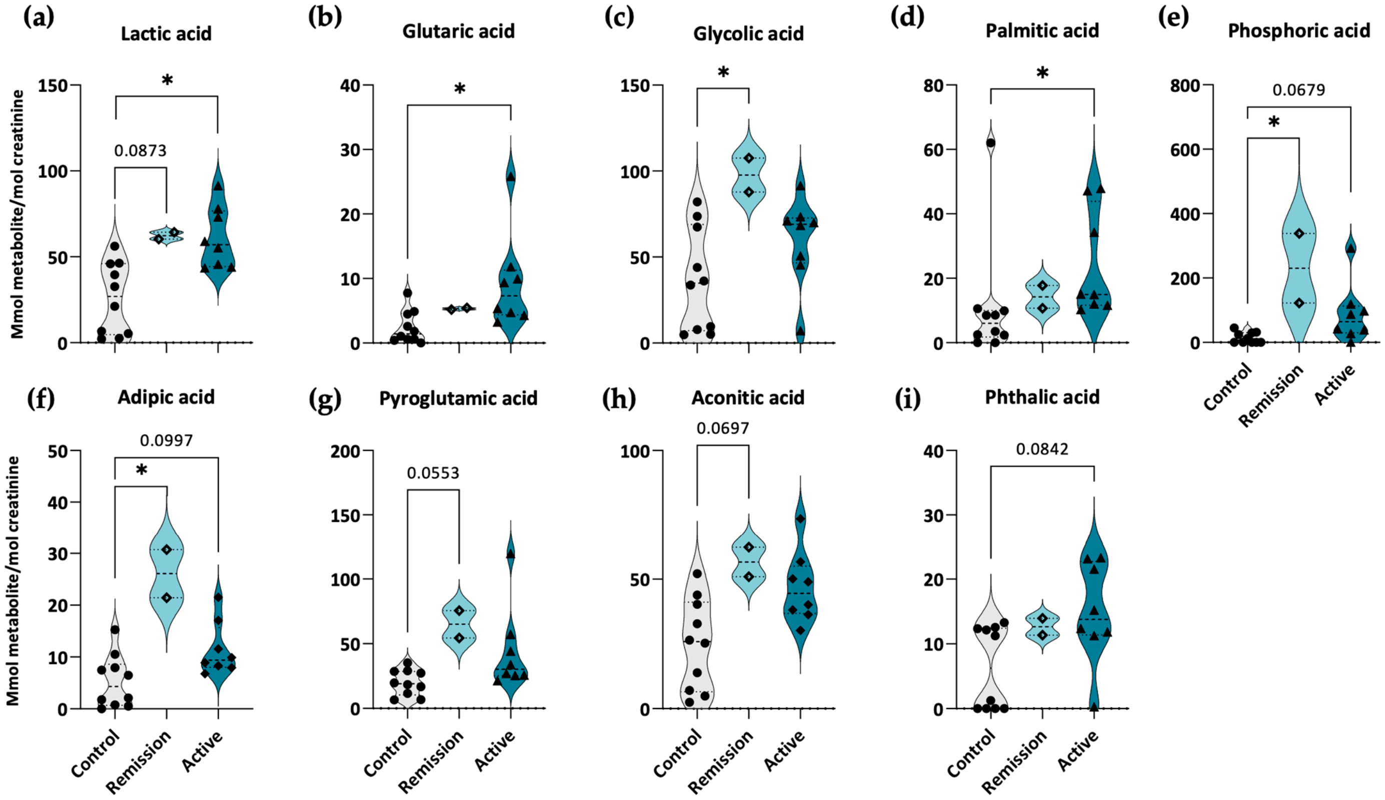

Figure S4: Specific metabolites associated with clinical stages in UC patients. Metabolites with VIP scores > 1 identified by PLS-DA comparing UC vs. C are shown stratified by disease activity (remission, mild, moderate) determined from endoscopic reports. Given the small sample size in some groups (n < 10), non-parametric analyses were applied directly. Therefore, group comparisons were performed using the Kruskal–Wallis test followed by Dunn's post-hoc test (*p<0.05).

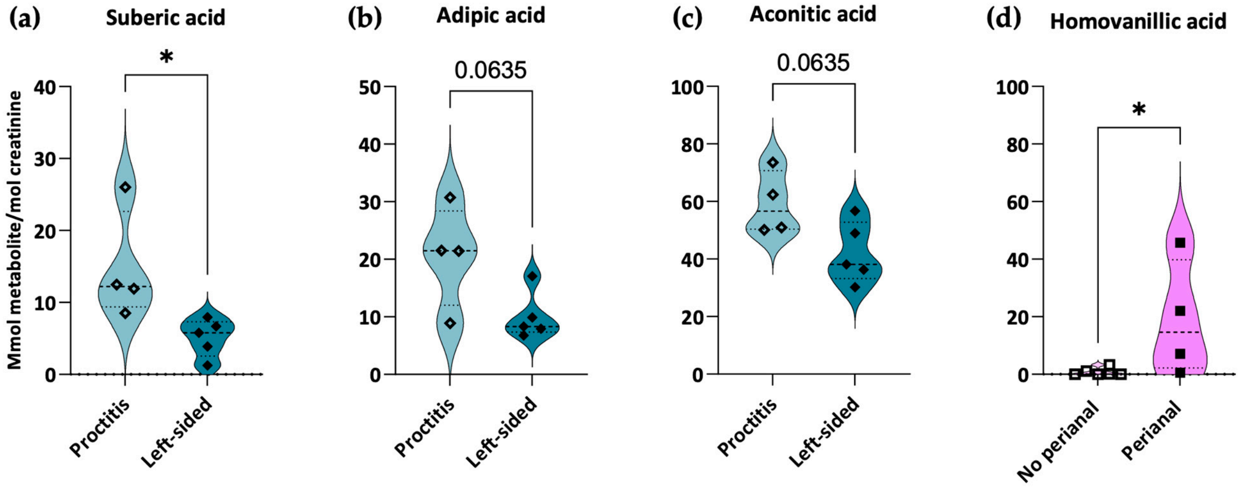

Figure S5: Specific metabolites associated with anatomical location of disease in ulcerative colitis and Crohn's disease patients. Metabolites with VIP scores > 1 identified by PLS-DA are shown stratified according to the anatomical location of disease involvement for UC (**a**-**c**) and CD (**d**), as determined by endoscopic reports. Comparisons were performed between UC patients according to disease extension (proctitis vs. left-sided colitis) and between CD patients according

to disease location (colonic, ileocolonic or perianal). Given the small sample size in some groups ($n < 10$), non-parametric analyses were applied directly. Therefore, group comparisons were performed using the Mann–Whitney U test was used (*$p < 0.05$). No multiple-testing adjustment was applied due to the exploratory nature of our study.

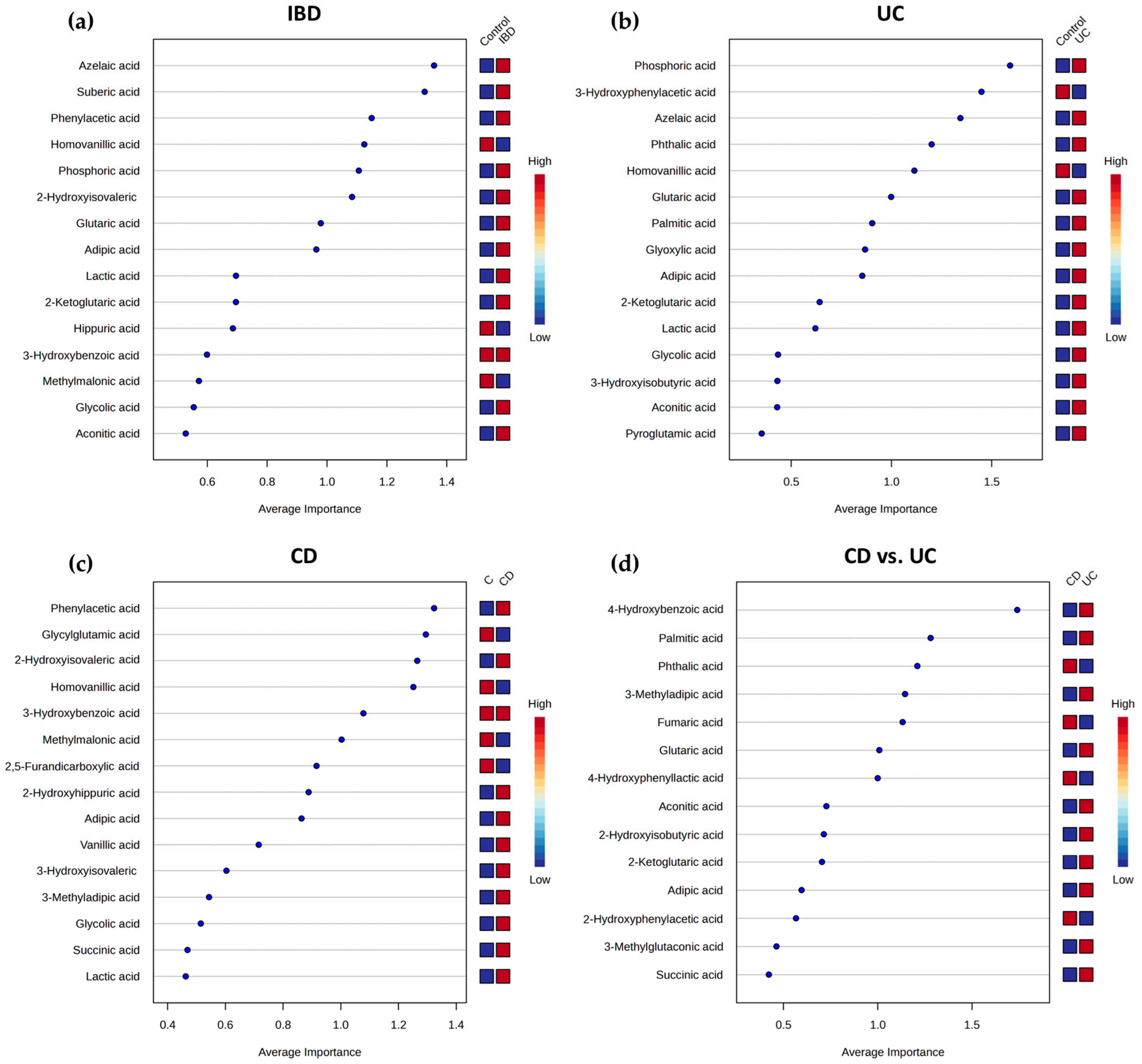

Figure S6: ROC-based analysis ranked metabolites as candidate biomarkers for IBD, UC, CD and CD vs UC**.** Urinary metabolites were ranked according to accurate biomarker model in ROC curves selected by PLS-DA. Ranking represents the discriminatory power of metabolites ordered from the most to the least significant metabolite concentration change. The comparative analyses were performed between the following groups: IBD vs. C (**a**), UC vs. C (**b**), CD vs. C (**c**), and CD vs. UC (**d**).
